# Risk factors for human leptospirosis following flooding: A meta-analysis of observational studies

**DOI:** 10.1371/journal.pone.0217643

**Published:** 2019-05-29

**Authors:** Cho Naing, Simon A. Reid, Saint Nway Aye, Norah Htet Htet, Stephen Ambu

**Affiliations:** 1 International Medical University, Kuala Lumpur, Malaysia; 2 Division of Tropical Health and Medicine, James Cook University, Townsville, Queensland, Australia; 3 School of Public Health, University of Queensland, Brisbane, Queensland, Australia; University of Colombo Faculty of Medicine, SRI LANKA

## Abstract

Leptospirosis is probably the most widespread zoonotic disease in the world especially in tropical countries. There has been an increase in individual studies, which assessed the frequency of leptospirosis in flood conditions. Some studies showed contact with floods was significantly associated with the occurrence of leptospirosis while other studies reported differently. The objective of this meta-analysis was to synthesize the evidence on the risk factors which are associated with human leptospirosis following flooding. We set up the inclusion criteria and searched for the original studies, addressing leptospirosis in human with related to flood in health-related electronic databases including PubMed, Embase, Ovid Medline, google scholar and Scopus sources. We used the terms ‘leptospirosis’, ‘flood’, ‘risk factor’ and terms from the categories were connected with “OR” within each category and by “AND” between categories. The initial search yielded 557 citations. After the title and abstract screening, 49 full-text papers were reviewed and a final of 18 observational studies met the pre-specified inclusion criteria. Overall, the pooled estimates of 14 studies showed that the contact with flooding was a significant factor for the occurrence of leptospirosis (pooled OR: 2.19, 95%CI: 1.48–3.24, *I*^*2*^:86%). On stratification, the strength of association was greater in the case-control studies (pooled OR: 4.01, 95%CI: 1.26–12.72, *I*^*2*^:82%) than other designs (pooled OR:1.77,95%CI:1.18–2.65, *I*^*2*^:87%). Three factors such as ‘being male’(pooled OR:2.06, 95%CI:1.29–2.83), the exposure to livestock animals (pooled OR: 1.95, 95%CI:1.26–2.64), the lacerated wound (pooled OR:4.35, 95%CI:3.07–5.64) were the risk factors significantly associated with the incidence of leptospirosis following flooding in the absence of within-study heterogeneity (*I*^*2*^: 0%). We acknowledge study limitations such as publication bias and type 2 statistical errors. We recommended flood control and other environmental modifications that are expected to reduce the risk of leptospiral infection, and a multi-sectoral effort to this aspect would have long-term benefits.

## Introduction

Leptospirosis is probably the most widespread zoonotic disease in the world [1] caused by corkscrew-shaped pathogenic bacteria *Leptospira interrogans* of the genus leptospira [2]. Human infection occurs through direct contact with the urine of animal reservoirs or through contact with contaminated soil or water [3]. The global burden of disease study estimated that approximately 2.9 million (1-25-4-54 million) Disability Adjusted Life Years were lost to leptospirosis. This is more than 70% of the global burden of cholera in that year. On stratification, males were approximately 80% and young adults aged 20–49 years were 52% of the total burden [4]. Although a widespread zoonotic in the world, it is particularly common in the tropics [1]. Clinical manifestations range from a mild form of illness including fever, chills and headache to life-threatening conditions (e.g., Weil disease), characterized by jaundice, renal impairment and hemorrhage [5]. The microagglutination titre (MAT) remains the definitive serological investigation technique in both humans and animals [6].

Leptospirosis as a human disease is the result of complex interactions between humans, animal reservoirs/carriers, and the environment where the bacteria can survive [7,8]. There remain significant gaps in our understanding of its transmission dynamics and of the trigger factors for disease outbreaks, including natural disasters. Flooding is the most common natural disaster both in developed and developing countries and is expected to occur with increasing frequency [9]. Flooding refers to the overflow of areas that are not normally submerged with water or a stream that has broken its normal confines or has accumulated due to lack of drainage [10].

There has been an increased in number of in individual studies which assessed the risk of leptospirosis in flood conditions. Some studies showed contact with floods was significantly associated with the occurrence of leptospirosis [11–13], while other studies reported differently [14,15]. In addition to the increased risk of leptospirosis after exposed exposure to flooding, socio-demographic (being male gender) and other predictors like sighting of rats were commonly reported in the individual studies. These individual studies varied in study population, study design, confirmation/ diagnostic methods of leptospirosis, among others. It is of an immense value to conduct a meta-analysis review, addressing the occurrence of human leptospirosis following flooding and identification of the associated risk factors. Taken together, the review questions were: what are the associated factors among human leptospirosis following flooding? If associated, what is the strength of association?

The objective was to synthesize the evidence on the risk factors which are associated with human leptospirosis following flooding. A better understanding of leptospirosis pertinent to flood exposure and its risk factors will enable the health policy planners to formulate more effective disease prevention and control strategies.

## Materials and methods

The current study adhered to the preferred reporting items for systematic reviews and meta- analyses (PRISMA) [16] (S1 Table).

### Search strategy

One investigator searched for the original studies, addressing leptospirosis in human with related to flood in health-related electronic database of PubMed, Embase (Ovid), Ovid Medline, Scopus sources, Google Scholar, the Latin American and Caribbean Health Sciences Literature (LILACS) and African Journals online (AJOL). Following the guidelines for the electronic search strategies [17], we searched the relevant studies using the following terms: ‘leptospirosis’, ‘flood’, ‘risk factor’. Terms from the categories were connected with “OR” within each category and by “AND” between categories. The PubMed search string used is provided in S2 Table. The search strategy was slightly adjusted according to the requirements of different databases. The search was limited to studies published in English until December 2018. The reference lists of the selected studies and relevant reviews were manually searched for any additional papers that were not captured by the electronic search.

### Selection criteria

Using the PICO format (population, participants or problem, intervention or exposure of interest, comparison and outcome) [18], the inclusion criteria of primary studies are shown in Table 1.

**Table 1:** Inclusion criteria.

Key point	Description
Participants	Individuals infected residing in endemic countries and had exposed to flooding, regardless of age and gender.
Exposure of interest	Studies which had explicitly revealed flooding as an exposure factor.
Comparison	1) Those individuals infected with leptospirosis following flooding and those who did not. 2) Those with associated predicators among human leptospirosis following flooding and those who did not.
Outcomes	1) Frequency of human leptospirosis following flooding. 2) Following flooding, frequency of human leptospirosis with associated risk factors. The strength of association was measured as odds ratio (OR) and its 95% confidence interval (CI). To be eligible, study must have reported data on at least one outcome.
Study design	Human observational studies investigating potential predictors for leptospirosis following flooding.

A laboratory-confirmed leptospirosis was as defined in the primary studies. These included the demonstration of a four-fold MAT rise between paired serum samples [5], a MAT reciprocal titre greater than 800 in one or more serum samples, or leptospires identified in blood or urine cultures by dark-field microscopy [19]. If more than one study presented data from the same participants, the most comprehensive study was included.

Studies were excluded, if they did not meet the inclusion criteria. Therefore, studies that included leptospirosis without revealing exposure to flooding were excluded. Also, reviews, case studies, editorials and conference abstracts were excluded.

### Data extraction

Two investigators individually screened the titles and abstracts, and then selected full-text articles, according to the inclusion criteria. The two investigators independently extracted information from each included study using a pre-tested data extraction form prepared for this meta-analysis. Information collected were first author, year of publication, year of study conducted, country, patient characteristics (e.g. age, gender), study characteristics (e.g. study design, sample size), flood description, risk factors (e.g. being male, sighting of rats) and the clinical characteristics (clinical form of leptospirosis, confirmation method). For any missing data, we planned to request from the corresponding author. However, this was not necessary. Disagreements between the two investigators were reached by consensus.

### Methodological quality assessment

The methodological quality of primary studies was assessed using the Newcastle-Ottawa Scale (NOS) [20]. This instrument uses a star system to evaluate methodological quality in relation to control of confounding variables, adequate sample size, minimised selection bias and clear definitions of exposures. Each article is given a score in number of stars from three perspectives: a) selection of the study groups (maximum: 4 stars), b) comparability of the study groups (maximum: 2 stars), and c) ascertainment of the outcome (maximum: 3 stars). Hence, a study could be achieved a maximum of nine scores. We used these scores only to facilitate the interpretation of the meta-analysis results, but not used as a criterion for inclusion or exclusion of articles. During these processes, any discrepancy between the two investigators was reached by consensus.

### Data analysis

For comparison of dichotomous data, we assumed that relative risk (RR) from cohort studies approximate OR from case-control studies [18]. As the incidence of human leptospirosis following flooding is not very high, a more conservative estimate of OR was used for the pooled analysis. If studies did not report RR or OR, we derived it from the raw data provided. Due to the availability of data, certain risk factors (e.g. gender difference, sighting of rats, behaviour related factor such as walking barefoot) that included in the primary studies were identified for this meta-analysis. To be included in pooled analysis, there must be at least three individual studies referring to a given risk factor. For the meta-analysis of flood-associated leptospirosis, we used inverse variance weighting, after transforming the estimates of each study into log OR and its standard error. For the meta-analysis of risk factors identification, we only included adjusted ORs from multivariate designs to minimise confounding in pooling of the effects. The heterogeneity within studies was assessed with the *I*^2^ test. A value of *I*^2^ >50% indicated substantial heterogeneity [18]. In the presence of substantial heterogeneity within studies, we used random-effect model for pooling of the effect sizes. To investigate the reasons for heterogeneity, subgroup analysis was done with study design and diagnostic methods of human leptospirosis (MAT or not). We assessed the publication bias by visualizing funnel plot asymmetry [21]. Data analyses were done by RevMan 5.3 (The Cochrane collaboration, Copenhagen), *R*. version 3.5.1 (The *R* Foundation) and *metan* package in STATA 15 (College Station, TX: StataCorp LLC).

## Results

**Fig 1:**
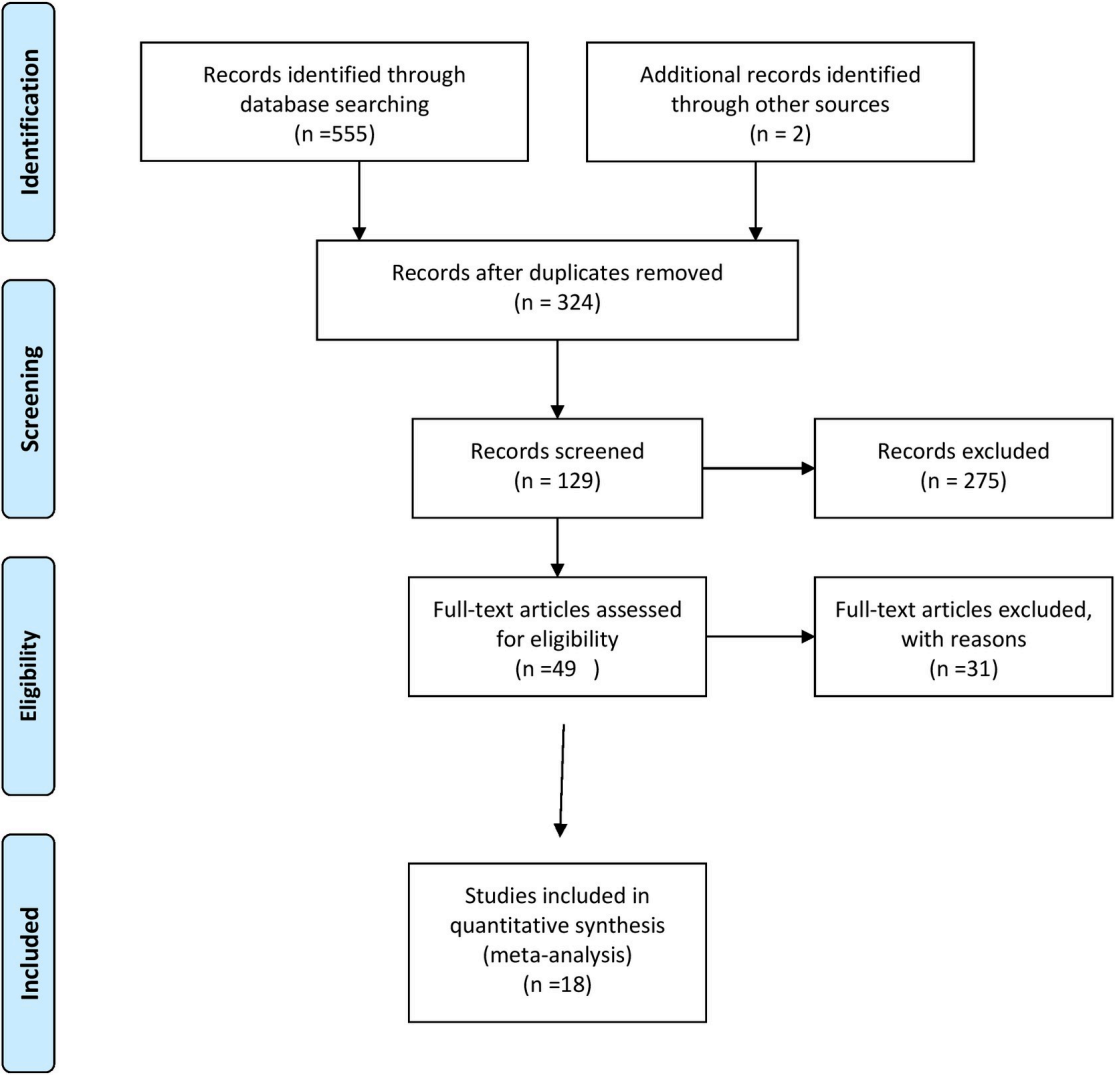
PRISMA flow diagram showing the study selection process.

A four-phase flow chart of the study selection process is illustrated in (Fig 1). The initial search yielded 557 citations. After the title and abstract screening, 49 full-text papers were reviewed and a final of eighteen studies met the pre-specified inclusion criteria [11,12, 14,15,19, 22–34]. Table 2 provides characteristics of these included studies. A summary of the thirty-one studies [5,7,13,35–62] are provided in S3 Table.

**Table 2:** Characteristics of studies included in the meta-analysis.

No	Author	Year of publication	Ref. no.	Year of study	Country of study	Study design	Age (mean ± SD in yr)	Males	Method of diagnosis^1^	Description of flood exposure	Incidence rate or cases vs controls	Quality grading
1	Trevejo	1998	22	1995	Nicaragua	CC	14.9	54.9%	MAT	following heavy flooding	51/2269	6
2	Ko	1999	19	1996	Brazil	CS	35·9 ± 15·2	79.3%	MAT	exposure to contaminated water	126/326	7
3	Barcellos	2001	23	1995	Brazil	CS^2^	NA	NA	NA	inside flood areas	35/83241	4
4	Sarkar	2002	24	2000	Brazil	CC	36.0 ± 14.2	89%	IgM (ELISA) + MAT	contact with flood water	101/157	8
5	Karande	2003	25	2000	Jamaica	CC	children	60.3%	IgM (ELISA)	h/o flood water contact	18/53	6
6	Leal-castellanos	2003	11	2000	Mexico	CS	≥15	22.6%	MAT	foot skin cuts or abrasion during flooding	441/1950	5
7	Dias	2006	14	2002	Brazil	CO	≥15 (74.6%)	41.7%	MAT	flooding in the household	172/1390	5
8	Gaynor	2007	26	2004	USA	CS	NA	NA	IgM EIA.	within 30 days of contact with flood water	1/48	5
9	Bharadwaj	2008	27	2006	India	CC	29.06 ±11.09	NA	IgM (ELISA)	contact of injured part with flood water	129 vs 253	7
10	Kawaguchi	2008	28	2006	Lao	CS	≥15	58.7%	MAT	flooding on one’s own property	97/406	6
11	Vanasco	2008	12	2000	Argentina	CC	32±16.2	87%	MAT	contact with contaminated surface water & flood	182/812	6
12	Keenan	2010	15	2006	Jamaica	CC	37.8	79.1%	IgG ELISA/ DST	home flooded	43 vs 89^3^	7
13	Dechet	2012	29	2005	Guyana	CO	NA	NA	MAT +RDT	extensive flooding	105/ 236	7
14	Agampodi	2014	30	2011	Sri Lanka	Prospective CO	40± 12	63.5%	qPCR	Following heavy rains and floods	96^4^	7
15	Chusri	2014	31	2013	Thailand	CC	≤60 (85.8%)	55.5%	IFAT/ MCAT	exposure to flood water	22/663	7
16	Felzemburg	2014	32	2003	Brazil	CO	≥15(88%)	60.7%	MAT	contact with flood water	35/1585	6
17	Lin	2015	33	2009–2010	Taiwan	longitudinal	58.42 ±12.73	33%	IgM/IgG	after flooding	2/288	6
18	Suwanpakdee	2015	34	2011	Thailand	CS	NA	NA	MAT/MCAT/ ELISA/ IFA/PCR/Culture	flooding	2129 in 2010; 3320 in 2011; 2213 in 2012	6

^1^: Method used to diagnose leptospirosis

^2^: spatial analysis

^3^: only numerator cases with no denominator population

^4^: probable case

CC: case-control design; CS: cross-sectional design; CO: Cohort design; DST: Dip-S-Ticks; IFAT: indirect immunofluorescent antibody test; Incidence: Incidence of leptospirosis (%);MAT:microscopic aggultination test; MCAT:microcapsule aggultination test; md: median and range; yr: year; wk: week

Of total eighteen studies, seven studies (39%) were case-control designs [12, 15, 22, 24, 25, 27, 31] and the remaining eleven studies (61%) were cohort or cross-sectional designs [11, 14, 19, 23, 26, 28–30, 32–34]. Of these, ten studies (55.6%) made confirmation of leptospirosis with the use of MAT (Table 2).

**Fig 2:**
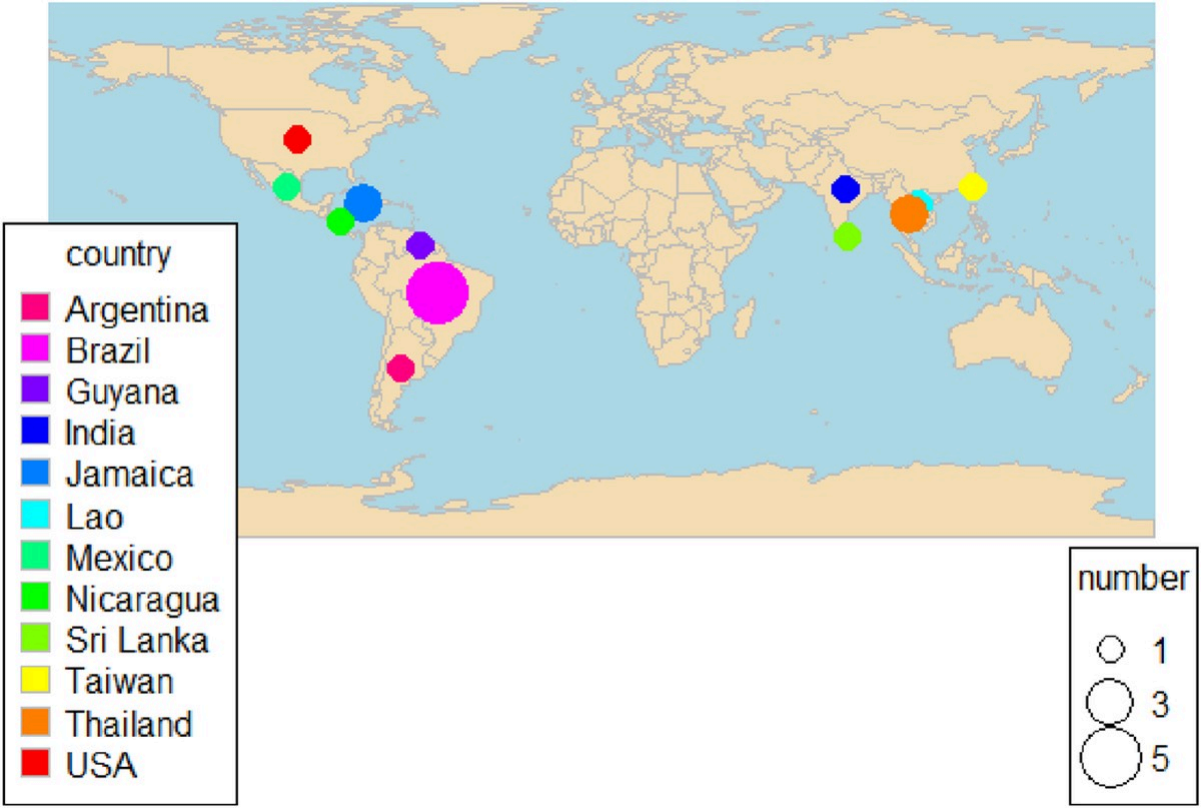
Distribution of the included studies.

Five studies (28%) included in this meta-analysis were conducted in Brazil [19, 23,24,14,32], while two studies (11%) each were in Jamaica [15,25] or Thailand [31, 34]. The remaining nine single studies were conducted in Argentina [12], India [27], Guyana [29], Lao [28], Mexico [11], Nicaragua [22], Sri Lanka [30], Taiwan [33] and the USA [26] (Fig 2). The studies included were published between 1995 and 2018. All these included studies revealed exposure to flood/flooding, albeit with variation in the descriptions. For instance, “home flooded”, “flooding in the household”, “flooding on one’s own property”, “flooding in the study district” or “inside flooded areas” [14,30,34]. Only one study indicated specifically about duration of stay in flood as “exposure to flood more or less than 3 hours per day” [31].

### Relationship with flooding

**Fig 3:**
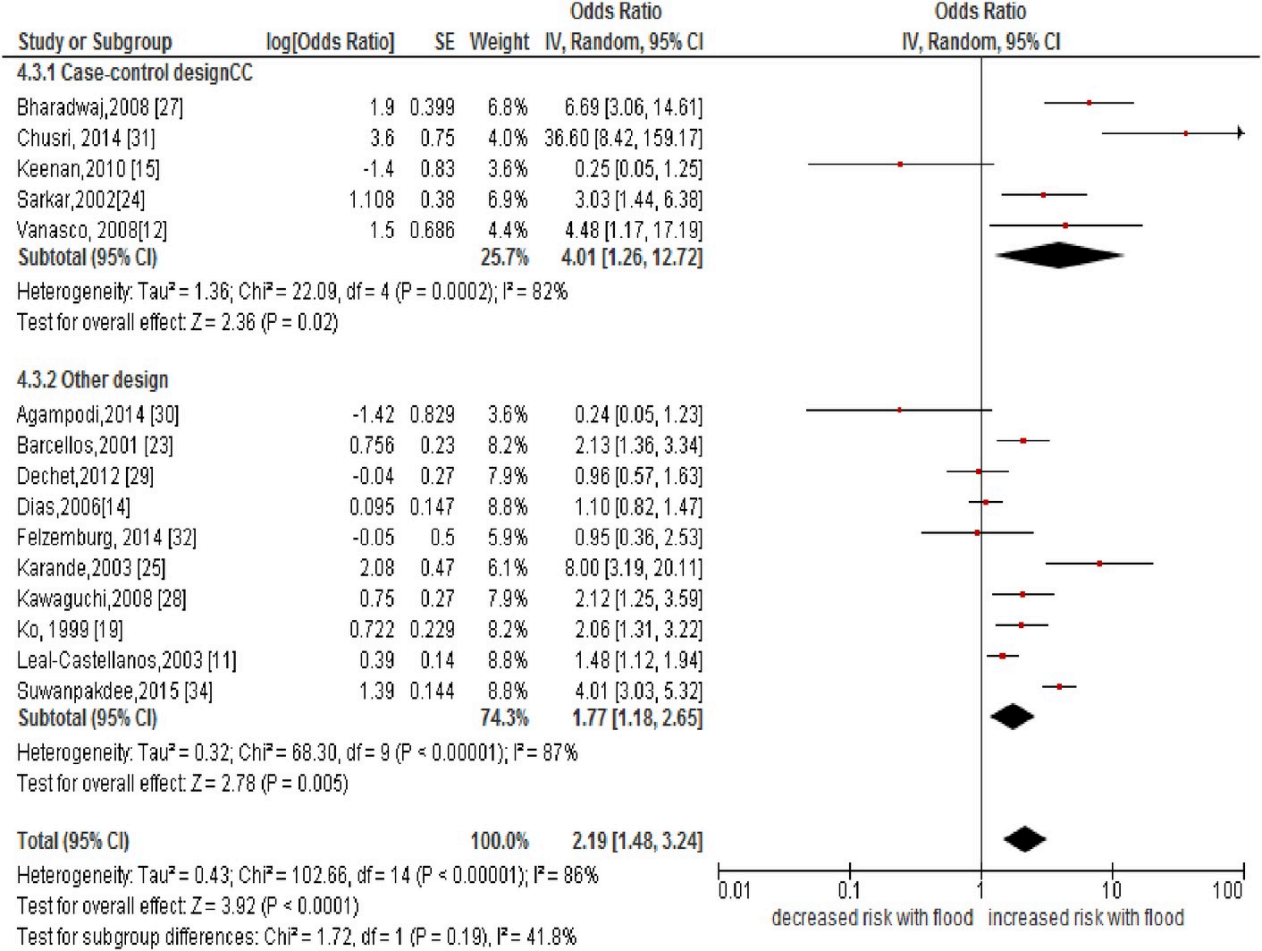
Forest plot showing an association between flooding and human leptospirosis.

Of eighteen studies included, fifteen [11, 12, 14,15,19,23–25,27–29,30–32,34] studies assessed the relationship between flooding and leptospirosis. Overall, the pooled estimates of fourteen studies showed that the contact with flooding was a significant factor for the occurrence of (increased) leptospirosis (pooled OR: 2.19, 95%CI: 1.48–3.24, *I*^*2*^:86%). On stratification, the strength of association was greater in the case-control studies (pooled OR: 4.01, 95%CI: 1.26–12.72, *I*^*2*^:82%) than other designs (pooled OR:1.77,95%CI:1.18–2.65, *I*^*2*^:87%). However, the confidence limit became wider in the pooled estimates of case control studies (Fig 3). This implied that the study design had only a minor impact on the effect estimation. When we retained studies that confirmed leptospirosis with MAT, there was only a negligible change in effect estimates (pooled OR: 2.18, 95%CI: 1.47–3.22, *I*^*2*^:86%) (S1 Fig); this implied that the diagnostic methods in these studies had only a very little impact on the effect estimation.

### Identification of risk factors

**Fig 4:**
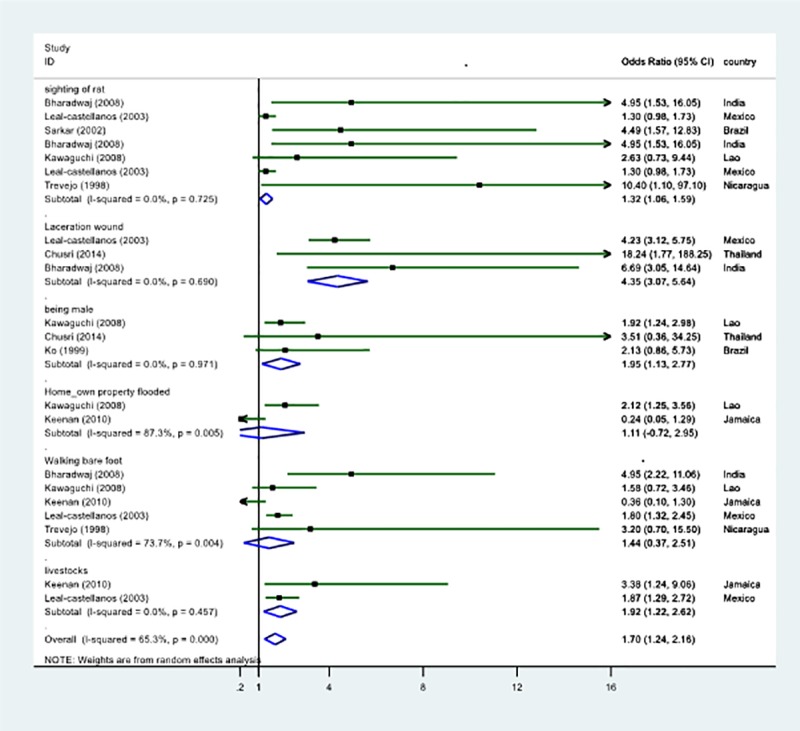
Associated risk factors for human leptospirosis following flooding.

The pooled estimates of five studies [11,22,24,27,28] showed that the sighting of rats was tended to be a significant risk factor for leptospirosis following flooding (pooled OR: 1.33, 95%CI:0.96–1.71, *I*^*2*^:0%). Also, the pooled estimates of three studies [11,27,31] showed that the lacerated wound was significantly associated with the occurrence of leptospirosis following flooding (pooled OR:4.35, 95%CI:3.07–5.64, *I*^*2*^:0%). Further, the pooled estimates of four studies [19,28,31,32] showed that being male was significantly associated with the occurrence of leptospirosis following flooding (pooled OR:2.06, 95%CI:1.29–2.83, *I*^*2*^:0%). Moreover, the pooled estimates of three studies [11,15,27] showed that exposure to livestock animals was associated with increased risk of leptospirosis cases (pooled OR: 1.95, 95%CI:1.26–2.64, *I*^*2*^:0%). Notably, three factors showed the increased associations with the risk of leptospirosis following flood in the absence of within-study heterogeneity (*I*^*2*^: 0%). A behaviour related factor such as ‘walking barefoot’ was reported in four studies [11,15,22,28], and the pooled estimate showed that it was not a risk for flood–related leptospirosis (pooled OR: 1.44, 95%CI:0.37–2.51, *I*^*2*^:74%) (Fig 4). There was evidence of publication bias in the current analysis (S2 Fig).

## Discussion

The current study containing eighteen observational studies from twelve endemic countries provides evidence on the effect of flooding and the associated risk factors for the occurrence of leptospirosis following flooding. The major observations of the current analysis are as follows;

1. A significant association between flooding and an increased risk of human leptospirosis was observed. This was more pronounced in the pooled analysis of case- control studies.
2. Following flooding, three factors such as being male, the lacerated wounds and exposure to livestock animals were significantly associated with the occurrence of leptospirosis, while sighting of rats was tended to be an increased risk.

Leptospirosis cases result from a combination of negative social and environmental conditions [23]. Earlier reviews addressed the occurrence of leptospirosis in various conditions [63, 64], while the current analysis focus solely on the flood related leptospirosis. This is because leptospiral infection would be more favourable in flooding as pathogenic Leptospira can survive freely in water and flooding prevents animal urine from being absorbed into the soil or evaporation. Hence, flooding would likely to be one of the main drivers for transmission by providing an optimal environment for survivability of leptospires [26]. Moreover, flooding may be possible in both rural settings and urban slums in many tropical countries. Evidence-based identification of risk factors is amenable to focused interventions in the peri-domiciliary environment. Environmental control of transmission may help to greatly reduce the incidence of leptospirosis [24].

Similar to the earlier reviews [63, 64], the present meta-analysis observed an increased risk of flood-related leptospirosis. A substantial within-study heterogeneity of the association might be partly due to differences in the definition of exposure to flooding or in the measurement of leptospirosis. Widespread flooding may offer potential risk of leptospirosis or lead to conditions favourable for outbreak leptospirosis when compared with non-flooded areas. This is because: (i) temporarily formed water accumulation is an ideal environment for proliferation of the leptospires contained in the urine [14]; (ii) there might be contaminated drinking water which is a possible transmission routes [4]; and (iii) there might be disruption of routine health facilities, allowing a poor/delayed health services. Natural disasters (flood in this case) cause sudden changes in the environment and also in human and animal behaviour. However, widespread transmission is limited at normal times due to a subtle balance between the determining factors, sudden changes in the environment and human behaviour create a situation conducive for the rapid transmission of leptospirosis resulting in outbreaks [65].

Peridomiciliary sighting of rats is feature of rat infestations and it was also a significant reservoir for leptospirosis [34]. Along this line, an empirical study had shown that *L*. *interrogans* (36% of positive samples) and *L*. *borgpetersenii* (56% of positive samples) species were widely distributed amongst rodent populations. Although these two most abundant leptospira species displayed different habitat requirements, they were confirmed as reservoirs for human leptospirosis [55]. During the flood or outbreak, specific rodent control programs could not be feasible to implement. A lack of proper rodent control during flood time had compounded to further transmission of infections.

Likewise, an earlier review [64], the present analysis documented the exposure to livestock as a significant risk for the occurrence of flood-related leptospirosis. Of note is that the associations documented in this review is merely a temporal relationship, but not a causation and the true source of infection could have been the rodents around animal farms rather than the animal themselves. Although live stocks livestock around residence was not reservoirs by themselves, rats were attracted to chicken feed and waste. A study had reported that 48% (519/1079) households in a slum community had practice of raising chickens [34]. Another epidemiologic study has reported a marginally increased risk (OR 1.15, 95%CI: 1.05–1.26) of seropositivity of leptospirosis and piggeries within 250 m and above house [7].

The increased risk of ‘being male’ might be due to the nature of their daily activities or occupational exposures likely to have more contact with leptospires [19,28] than the females. Laceration wound was defined as a wound caused by tearing of the skin deeper than the epidermis and through the soft tissue [31]. An association of a presence of laceration wound and leptospirosis was reported in the current analysis and this might be explained that cuts or abrasions in the skin or mucous membranes could facilitate entry of leptospira spirochetes through the mechanisms of counter-clockwise rotation of the periplasmic flagella [66]. This would be more favourable in flooding as pathogenic leptospira can live freely in water. This implied that leptospira multiplied in the walking paths where water remained in the flooded areas [27]. The ability to adhere to host tissue via adhesion proteins is also thought to be a necessary component of leptospire pathogenicity [67].

## Study limitations

There are some limitations in the present meta-analysis. Leptospirosis is considered to be widespread in many tropical countries. The limited number of studies to the limited countries made a limited geographic representativeness. Studies in non-English language were not included. This concern was supported by the presence of publication bias observed in the current analysis. Flooding results from the interaction of many factors including rainfall, surface run-off, sea level, catchment size and local topography [7]. Besides flooding as a factor, several other factors can affect the isolation of leptospira from water and soil, such as pH, temperature, characteristics of water and soil and the presence of animals that are considered as reservoirs of leptospira. The lack of significance in the remaining risk factors (e.g. barefooted walking) might partly be due to the relatively small number of studies with small sample sizes, which was an issue of Type II statistical error. Hence, the association between flooding and the occurrence of human leptospirosis observed in the present analysis could be interpreted in the light of these issues. Nevertheless, our analysis used the adjusted ORs controlling common factors for the strength of associations, and this made a confidence in our estimates.

## Public health implications

Leptospiral infections cluster in the flooded areas was documented in the present analysis. Flooding is related to peri-domiciliary infrastructure deficiencies (inadequate rainwater drainage with silt and refuse, blockage of drainage system). Considering that leptospira can survive for weeks to months in fresh water, and are efficiently carried and disseminated by water [60] (e.g. flooding in this case). The identified risk factors are conditions that are potentially correctable not by individuals, but at the municipal and community level [24]. Hence, better control strategies and immediate response system involving multisectoral approaches including health and non-health sectors (e.g. land development, rural/urban settlement, animal husbandry, municipalities) would have a significant impact on the reduction of leptospirosis transmission amongst the targeted populations. For example, improving sanitation in slum communities and equitable access to improved sanitation [34] would reduce rat infestations and further exposure to an environment contaminated by the urine of rodent reservoirs.

Health education on proper garbage system in households and work stations would reduce source of food for rodents and further reduction in rodent infestations. Also, easily adopted measures as an example of protective wearing (e.g. avoidance of barefoot walking, protective clothing at work) should be promoted among high risk subjects [24]. This would prevent/reduce the occurrences of cuts/ abrasion in skin and subsequent entry of spirochetes. Local flooding is documented as an important role in the transmission of leptospirosis. Therefore, flood control and other environmental modifications are expected to reduce the risk of leptospiral infection [28] and a multi-sectoral effort to this aspect would have long term benefits.

As severe manifestations of leptospirosis are characterized by high mortality, point-of- care clinicians’ index of clinical suspicion for leptospirosis in early stage is crucially important. This heightened awareness, and the availability of diagnostic testing will improve surveillance for sporadic cases of leptospirosis and help prevent, detect, and control future outbreaks [26].

## Conclusions

Findings suggest that flooding was associated with the risk of occurrence of leptospirosis in endemic countries. Future public health interventions such as implementation of disease prevention measures including rodent control programs and health education should be valuable for preventing exposure to animal urine and subsequent leptospirosis. These findings may serve as evidence-based information for the national health planners for better formulation of a package of emergency disease control strategy containing leptospirosis. To substantiate the findings, future well designed prospective studies complement to proper health care services during flooding are required.

## Supporting information

S1 Table PRISMA checklist. (DOC)

S2 Table The PubMed search string. (DOC)

S3 Table Excluded studies and reasons for exclusion. (DOC)

S1 Fig An association between flooding and the risk of human leptospirosis confirmed with MAT. (TIF)

S2 Fig Funnel plot showing publication bias. (TIF)

## References

[1] Gubler DJ, Reiter P, Ebi KL, Yap W, Nasci R, Patz JA. Climate variability and change in the United States: potential impacts on vector- and rodent-borne diseases. Environmental Health Perspectives. 2001; 109(Suppl 2): 223–233. 11359689 10.1289/ehp.109-1240669 PMC1240669

[2] World Health Organization (WHO). Human leptospirosis: guidance for diagnosis, surveillance and control. Geneva: 2003. Available from:https://apps.who.int/iris/handle/10665/42667.

[3] Bharti A, Nally J, Ricaldi J, Matthias M, Diaz M, Lovett M, et al Leptospirosis: a zoonotic disease of global importance. Lancet Infect Dis. 2003; 3:757–771. 14652202 10.1016/s1473-3099(03)00830-2

[4] Torgerson PR, Hagan JE, Costa F, Calcagno J, Kane M, Martinez-Silveira MS, et al Global burden of leptospirosis: estimated in terms of disability adjusted life years. PLoS Negl Trop Dis. 2015; 9(10): e0004122 10.1371/journal.pntd.0004122 26431366 PMC4591975

[5] Levett PN. Leptospirosis. Clin Microbiol Rev.2001; 14:296–326. 10.1128/CMR.14.2.296-326.2001 11292640 PMC88975

[6] Matono T, Kutsuna S, Koizumi N, Fujiya Y, Takeshita N, Hayakawa K, et al Imported flood-related leptospirosis from Palau: awareness of risk factors leads to early treatment. J Travel Med. 2015;22(6):422–424. 10.1111/jtm.12241 26503094

[7] Lau CL, Smythe LD, Craig SB, Weinstein P. Trans R Soc Trop Med Hyg. 2010; 104: 631–638. 10.1016/j.trstmh.2010.07.002 20813388

[8] Guernier V, Goarant C, Benschop J, Lau CL. A systematic review of human and animal leptospirosis in the Pacific Islands reveals pathogen and reservoir diversity. PLoS Negl Trop Dis. 2018; 12(5): e0006503 10.1371/journal.pntd.0006503 29758037 PMC5967813

[9] Patz JA, Kovats RS. Hotspots in climate change and human health. BMJ. 2002; 325:1094–1098. 10.1136/bmj.325.7372.1094 12424173 PMC1124582

[10] Keim ME. Building human resilience: the role of public health preparedness and response as an adaptation to climate change. Am J Prev Med. 2008; 35:508–516. 10.1016/j.amepre.2008.08.022 18929977

[11] Leal-Catellanos CB, Garcia-Suarez R, Gonzalez-Figueroa E, Fuentes-Allen JL, Escobedo-De La Pena J, et al. Risk factors and the prevalence of leptospirosis infection in a rural community of Chiapas, Mexico. Epidemiology and Infection. 2003; 131: 1149–1156. 14959783 10.1017/s0950268803001201 PMC2870065

[12] Vanasco NB, Schmeling MF, Lottersberger J, Costa F, Ko AI, Tarabla HD. Clinical characteristics and risk factors of human leptospirosis in Argentina (1999–2005). Acta Tropica. 2008; 107(3):255–258. 10.1016/j.actatropica.2008.06.007 18671932

[13] Wijerathne KB, Senevirathna EM. Identify the risk for leptospirosis disease during flooding periods (Special reference to Medirigiriya Divisional Secretariat Division in Polonnaruwa district). Procedia Engineering. 2018; 212:101–108.

[14] Dias JP, Teixeira MG, Costa MC, Mendes CM, Guimarães P, Reis MG, et al Factors associated with Leptospira sp infection in a large urban center in North-eastern Brazil. Revista da Sociedade Brasileira de Medicina Tropical. 2007, 40(5):499–504. 17992402 10.1590/s0037-86822007000500002

[15] Keenan J, Ervin G, Aung M, McGwin JG, Jolly P. Risk factors for clinical leptospirosis from Western Jamaica. Am J Trop Med Hyg 2010;83(3):633–636. 10.4269/ajtmh.2010.09-0609 20810831 PMC2929062

[16] Moher D, Liberati A, Tetzlaff J, Altman DG. Preferred reporting items for systematic reviews and meta-analyses: the PRISMA statement. BMJ. 2009; 339: b2535 10.1136/bmj.b2535 19622551 PMC2714657

[17] Sampson M, McGowan J, Cogo E, Grimshaw J, Moher D, Lefebvre C. An evidence-based practice guideline for the peer review of electronic search strategies. J Clin Epidemiol. 2009; 62(9):944–952. 10.1016/j.jclinepi.2008.10.012 19230612

[18] Higgins JPT, Green S, ed. Cochrane Handbook for Systematic Reviews of Interventions. Version 5.1.0 (updated March 2011). The Cochrane Collaboration; 2011.

[19] Ko AI, Reis MG, Dourado CMR, Johnson Jr WD, Riley LW and Salvador Leptospirosis Study Group. Urban epidemic of severe leptospirosis in Brazil. The Lancet. 1999; 354(9181), 820–825. 10.1016/s0140-6736(99)80012-9 10485724

[20] Wells GA, Shea B, O’Connell D, Peterson J, Welch V, Losos M, et al. The Newcastle-Ottawa Scale (NOS) for assessing the quality of non-randomised studies in meta-analyses. Ottawa 2011 Hospital Research Institute. Available from: www.ohri.ca/programs/clinical_epidemiology/oxford.htm

[21] Sterne JA, Egger M. Funnel plots for detecting bias in meta-analysis: guidelines on choice of axis. J Clin Epidemiol. 2001; 54:1046–1055. 11576817 10.1016/s0895-4356(01)00377-8

[22] Trevejo RT, Rigau-Perez JG, Ashford DA, McClure EM, Jarquin-Gonzalez C, Amador JJ, et al Epidemic leptospirosis associated with pulmonary hemorrhage–Nicaragua, 1998 J Infect Dis 178:1457–1463. 10.1086/314424 9780268

[23] Barcellos C, Sabroza PC. The place behind the case: leptospirosis risks and associated environmental conditions in a flood-related outbreak in Rio de Janeiro. Cadernos de Saude Publica. 2001;17S:59–67. 10.1590/s0102-311x2001000700014 11426266

[24] Sarkar U, Nascimento SF, Barbosa R, Martins R, Nuevo H, Kalofonos I, et al Population based case-control investigation of risk factors for leptospirosis during an urban epidemic. Am J Trop Med Hyg 2002; 66(5):605–610. 12201599 10.4269/ajtmh.2002.66.605

[25] Karande S, Bhatt M, Kelkar A, Kulkarni M, De A, Varaiya A. An observational study to detect leptospirosis in Mumbai, India, 2000. Archives of disease in childhood. 2003;88(12):1070–1075. 10.1136/adc.88.12.1070 14670771 PMC1719391

[26] Gaynor K, Katz AR, Park SY, Nakata M, Clark TA, Effler PV. Leptospirosis on Oahu: an outbreak associated with flooding of a university campus. Am J Trop Med Hyg. 2007;76(5):882–886. 17488909

[27] Bhardwaj P, Kosambiya JK, Desai VK. A case-control study to explore the risk factors for acquisition of leptospirosis in Surat city, after flood. Indian J Med Sci 2008; 62:431–438. 19265232

[28] Kawaguchi L, Sengkeopraseuth B, Tsuyuoka R, Koizumi N, Akashi H, Vongphrachanh P, et al Seroprevalence of leptospirosis and risk factor analysis in flood-prone rural areas in Lao PDR. Am J Trop Med Hyg. 2008;78(6):957–961. 18541776

[29] Dechet AM, Parsons M, Rambaran M, Mohamed-Rambaran P, Florendo-Cumbermack A, Persaud S, et al Leptospirosis outbreak following severe flooding: a rapid assessment and mass prophylaxis campaign; Guyana, Jan-Feb 2005 PLoS ONE. 2012: 7(7): e39672 10.1371/journal.pone.0039672 PMC3392270 22808049

[30] Agampodi SB, Dahanayaka NJ, Bandaranayaka AK, Perera M, Priyankara S, Weerawansa P, et al Regional differences of leptospirosis in Sri Lanka: observations from a flood-associated outbreak in 2011. PLoS Negl Trop Dis. 2014;8(1): e2626 10.1371/journal.pntd.0002626 24454971 PMC3894175

[31] Chusri S, McNeil EB, Hortiwakul T, Charernmak B, Sritrairatchai S, Santimaleeworagun W, et al Single dosage of doxycycline for prophylaxis against leptospiral infection and leptospirosis during urban flooding in southern Thailand: A non-randomized controlled trial. J Infect Chemother. 2014; 20(11):709–715. 10.1016/j.jiac.2014.07.016 25172777

[32] Felzemburgh RD, Ribeiro GS, Costa F, Reis RB, Hagan JE, Melendez AX, et al Prospective study of leptospirosis transmission in an urban slum community: role of poor environment in repeated exposures to the Leptospira agent. PLoS Negl Trop Dis. 2014;8(5): e2927 10.1371/journal.pntd.0002927 24875389 PMC4038618

[33] Lin CY, Chen TC, Dai CY, YU ML, Lu PL, Yen JH, et al Serological investigation to identify risk factors for post-flood infectious diseases: a longitudinal survey among people displaced by Typhoon Morakot in Taiwan. BMJ Open. 2015;5: e007008 10.1136/bmjopen-2014-007008 25976763 PMC4442151

[34] Suwanpakdee S, Kaewkungwal J, White LJ, Asensio N, Ratanakorn P, Singhasivanon P, et al Spatio-temporal patterns of leptospirosis in Thailand: is flooding a risk factor? Epidemiol Infect. 2015; 143: 2106–2115. 10.1017/S0950268815000205 25778527 PMC4462160

[35] Bovet P, Yersin C, Merien F, Davis CE, Perolat P. Factors associated with clinicalleptospirosis: a population-based case-control study in the Seychelles (Indian Ocean). Int J Epidemiol. 1999;28(3):583–590. 10.1093/ije/28.3.583 10405868

[36] Kupek E, de Sousa Santos Faversani MC, de Souza Philippi JM. The relationship between rainfall and human leptospirosis in Florianopolis, Brazil, 1991–1996. Braz J Infect Dis. 2000:4: 131–134. 10934496

[37] Tangkanakul W, Tharmaphornpil P, Plikaytis BD, Bragg S, Poonsuksombat D, Choomkasien P, et al Risk factors associated with leptospirosis in north-eastern Thailand,1998. Am J Trop Med Hyg. 2000;63(3): 204–208. 11388516 10.4269/ajtmh.2000.63.204

[38] Jena AB, Mohanty KC, Devadasan N. An outbreak of leptospirosis in Orissa, India: the importance of surveillance. Trop Med Int Health. 2004;9(9): 1016–1021. 10.1111/j.1365-3156.2004.01293.x 15361116

[39] Schwartz J, Samet JM, Patz JA. Hospital admissions for heart disease: the effects of temperature and humidity. Epidemiology. 2004;15(6): 755–761. 15475726 10.1097/01.ede.0000134875.15919.0f

[40] Yimer E, Koopman S, Messele T, Wolday D, Newayeselassie B, Gessese N. Human leptospirosis, in Ethiopia: a pilot study in Wonji. Ethiop J Health Dev. 2004;18(1): 48–51.

[41] Hadad E, Pirogovsky A, Bartal C, Gilad J, Barnea A, Yitzhaki S, et al An outbreak of leptospirosis among Israeli troops near the Jordan River. Am J Trop Med Hyg. 2006;74(1): 127–131. 16407357

[42] Maskey M, Shastri JS, Saraswathi K, Surpam R, Vaidya N. Leptospirosis in Mumbai:post-deluge outbreak 2005. Indian J Med Microbiol. 2006;24(4): 337–338. 17185871 10.4103/0255-0857.29413

[43] Yanagihara Y, Villanueva SY, Yoshida S, Okamoto Y, Masuzawa T. Current status of leptospirosis in Japan and Philippines. Comp Immunol Microbiol Infect Dis. 2007;30: 399–413. 10.1016/j.cimid.2007.05.003 17614131

[44] Reis RB, Ribeiro GS, Felzemburgh RDM, Santana FS, Mohr S, Melendez AO, et al Impact of environment and social gradient on leptospira infection in urban slums. PLoS Negl Trop Dis. 2008:2(4): e228 10.1371/journal.pntd.0000228 18431445 PMC2292260

[45] Mathur M, De A, Turbadkar D. Leptospirosis outbreak in 2005: LTMG hospital experience. Indian J Med Microbiol. 2009;27(2): 153–155. 10.4103/0255-0857.49431 19384041

[46] Desai S, van Treeck U, Lierz M, Espelage W, Zota L, Sarbu A, et al Resurgence of field fever in a temperate country: an epidemic of leptospirosis among seasonal strawberry harvesters in Germany in 2007. Clin Infect Dis. 2009;48(6):691–697. 10.1086/597036 19193108

[47] Stern EJ, Galloway R, Shadomy SV, Wannemuehler K, Atrubin D, Blackmore C, et al Outbreak of leptospirosis among adventure race participants in Florida, 2005. Clin Infect Dis. 2010; 50(6):843–849. 10.1086/650578 20146629

[48] Amilasan TA, Ujii M, Suzuki M, Salva E, Belo MC, Koizumi N, et al Outbreak of leptospirosis after flood, the Philippines, 2009. Emerg Infect Dis. 2012;18(1):91–94. 10.3201/eid1801.101892 22257492 PMC3310081

[49] Chen MJ, Lin CY, Wu YT, Wu PC, Lung SC, Su HJ. Effects of extreme precipitation to the distribution of infectious diseases in Taiwan, 1994–2008. PLoS One. 2012;7(6):e34651 10.1371/journal.pone.0034651 22737206 PMC3380951

[50] Smith JKG, Young MM, Wilson KL, Craig SB. Leptospirosis following a major flood in Central Queensland, Australia. Epidemiol Infect First View. 2012; 141(3): 585–590. 10.1017/S0950268812001021 PMC9151845 22625176

[51] Bello S, Rodriguez M, Paredes A, Mendivelso F, Walteras D, Rodriguez F, et al Comportamiento de la vigilancia epidemiológica de la leptospirosis humana en Colombia, 2007–2011. Biomedica 2013;33(Supl.1):153–160. 24652259

[52] Miyazato KE, Fonseca AL, Caputto LZ, Rocha KC, Azzalis LA, Junqueira VB, et al Incidence of leptospirosis infection in the East zone of Sao Paulo City, Brazil. Int Arch Med. 2013;6(1):23 10.1186/1755-7682-6-23 23672682 PMC3682875

[53] Cosson JF, Picardeau M, Mielcarek M, Tatard C, Chaval Y, Suputtamongkol Y, et al Epidemiology of Leptospira transmitted by rodents in Southeast Asia. PLoS Negl Trop Dis 2014;8(6): e2902 10.1371/journal.pntd.0002902 24901706 PMC4046967

[54] Munoz-Zanzi C, Mason M, Encina C, Gonzalez M, Berg S. Household characteristics associated with rodent presence and Leptospira infection in rural and urban communities from Southern Chile. Am J Trop Med Hyg. 2014; 90(3): 497–506. 10.4269/ajtmh.13-0334 24445209 PMC3945004

[55] Kamath R, Swain S, Pattanshetty S, Nair NS. Studying risk factors associated with human leptospirosis. J Glob Infect Dis. 2014;6(1): 3–9. 10.4103/0974-777X.127941 24741223 PMC3982353

[56] Benacer D, Thong KL, Min NC, Verasahib KB, Galloway RL, Hartskeerl RA, et al Epidemiology of human leptospirosis in Malaysia, 2004–2012. Acta Tropica. 2016;157: 162–168. 10.1016/j.actatropica.2016.01.031 26844370

[57] Dreyfus A, Dyal JW, Pearson R, Kankya C, Kajura C, Alinaitwe L, et al Leptospira seroprevalence and risk factors in health centre patients in Hoima District, Western Uganda. PLoS Negl Trop Dis. 2016;10(8): e0004858 10.1371/journal.pntd.0004858 27487398 PMC4972303

[58] Lau CL, Watson CH, Lowry JH, David MC, Craig SB, Wynwood SJ, et al. (2016) Human leptospirosis infection in Fiji: An eco-epidemiological approach to identifying risk factors and environmental drivers for transmission. PLoS Negl Trop Dis. 2016:10(1): e0004405 10.1371/journal.pntd.0004405 26820752 PMC4731082

[59] Fajriyah SN, Udiyono A, Saraswati LD. Environmental and risk factors of leptospirosis: a spatial analysis in Semarang City. In IOP conference series: Earth Environ Sci. 2017;55 (1). 10.1088/1755-1315/55/1/012013

[60] Regmi L, Pandey K, Malla M, Khanal S, Pandey BD. Sero-epidemiology study of leptospirosis in febrile patients from Terai region of Nepal. BMC Infect Dis. 2017;17(1):628 10.1186/s12879-017-2733-x 28923024 PMC5604353

[61] Matsushita N, Ng CF, Kim Y, Suzuki M, Saito N, Ariyoshi K, et al The non-linear and lagged short-term relationship between rainfall and leptospirosis and the intermediate role of floods in the Philippines. PLoS Negl Trop Dis. 2018;12(4): e0006331 10.1371/journal.pntd.0006331 29659576 PMC5919665

[62] Radi MF, Hashim JH, Jaafar MH, Hod R, Ahmad N, Nawi AB, et al Leptospirosis outbreak after the 2014 major flooding event in Kelantan, Malaysia: a spatial-temporal analysis. Am J Trop Med Hyg. 2018;95(5): 1281–195. 10.4269/ajtmh.16-0922 PMC5953347 29532771

[63] Brown L, Murray V. Examining the relationship between infectious diseases and flooding in Europe. A systematic literature review and summary of possible public health interventions. Disaster Health. 2013:1(2): 117–127. 10.4161/dish.25216 28228994 PMC5314884

[64] Mwachui MA, Crump L, Hartskeerl R, Zinsstag J, Hattendorf J. Environmental and Behavioural determinants of leptospirosis transmission: a systematic review. PLoS Negl Trop Dis. 2015; 9(9): e0003843 10.1371/journal.pntd.0003843 26379035 PMC4574979

[65] Sehgal SC, Sugunan AP, Vijayachari P. Outbreak of leptospirosis after the cyclone in Orissa. The National medical journal of India. 2002;15(1):22–23. 11855587

[66] Wolgemuth CW. Flagellar motility of the pathogenic spirochetes. Semin Cell Dev Biol. 2015;46: 104–112. 10.1016/j.semcdb.2015.10.015 26481969 PMC4994469

[67] Cullen PA, Haake DA, Adler B. Outer membrane proteins of pathogenic spirochetes. FEMS Microbiol Rev. 2004;28(3):291–318. 10.1016/j.femsre.2003.10.004 15449605 PMC2666356

