# Supplementary material for: Risk factors for human leptospirosis following flooding: A meta-analysis of observational studies
Source: PLoS One. 2019 May 29;14(5):e0217643. doi: 10.1371/journal.pone.0217643 (PMC6541304; doi:10.1371/journal.pone.0217643)
Supplement: S2 Table — (DOC) [file pone.0217643.s002.doc]

S2 Table. The PubMed search string

| (((((((((((((("floods"[MeSH Terms] OR "floods"[All Fields] OR "flood"[All Fields]) OR ("implosive therapy"[MeSH Terms] OR ("implosive"[All Fields] AND "therapy"[All Fields]) OR "implosive therapy"[All Fields] OR "flooding"[All Fields] OR "floods"[MeSH Terms] OR "floods"[All Fields])) AND ("leptospirosis"[MeSH Terms] OR "leptospirosis"[All Fields])) OR lepto[All Fields]) OR ("weil disease"[MeSH Terms] OR ("weil"[All Fields] AND "disease"[All Fields]) OR "weil disease"[All Fields] OR ("weil's"[All Fields] AND "disease"[All Fields]) OR "weil's disease"[All Fields])) NOT ("case"[All Fields] AND strudy[All Fields])) NOT ("economics"[MeSH Terms] OR "economics"[All Fields] OR "economic"[All Fields])) NOT modelling[All Fields]) NOT ("models, animal"[MeSH Terms] OR ("models"[All Fields] AND "animal"[All Fields]) OR "animal models"[All Fields] OR ("animal"[All Fields] AND "model"[All Fields]) OR "animal model"[All Fields])) AND ("humans"[MeSH Terms] OR "humans"[All Fields] OR "human"[All Fields])) NOT ("pharmaceutical preparations"[MeSH Terms] OR ("pharmaceutical"[All Fields] AND "preparations"[All Fields]) OR "pharmaceutical preparations"[All Fields] OR "drugs"[All Fields])) NOT ("pharmacology"[Subheading] OR "pharmacology"[All Fields] OR "pharmacology"[MeSH Terms])) NOT ("clinical trial"[All Fields] OR "clinical trials as topic"[MeSH Terms] OR "clinical trial"[All Fields])) NOT ("therapeutics"[MeSH Terms] OR "therapeutics"[All Fields] OR "therapeutic"[All Fields])) AND ("risk factors"[MeSH Terms] OR ("risk"[All Fields] AND "factors"[All Fields]) OR "risk factors"[All Fields] OR ("risk"[All Fields] AND "factor"[All Fields]) OR "risk factor"[All Fields]) |
| --- |
