# Supplementary material for: Risk factors for human leptospirosis following flooding: A meta-analysis of observational studies
Source: PLoS One. 2019 May 29;14(5):e0217643. doi: 10.1371/journal.pone.0217643 (PMC6541304; doi:10.1371/journal.pone.0217643)
Supplement: S3 Table — (DOC) [file pone.0217643.s003.doc]

S3 Table. Excluded studies and reasons for exclusion

| Sr. No | Study | Ref.no | Reason |
| --- | --- | --- | --- |
| 1. | Bovet, 1999 | 35 | Not flood as a risk factor |
| 2. | Kupek,2000 | 36 | No data with flood exposure. |
| 3 | Tangkanakul,2000 | 37 | No description on flood |
|  | Jena, 2004 | 38 | No description of flood |
|  | Schwartz,2004 | 39 | Not flood as a risk factor |
|  | Yimer,2004 | 40 | Autopsy samples included. |
|  | Hadad,2006 | 41 | No description on flood |
|  | Maskey,2006 | 42 | Diagnostic accuracy |
|  | Yanagihara,2007 | 43 | Case series |
|  | Reis,2008 | 44 | No data with flood exposure. |
|  | Mathur, 2009 | 45 | not a study for risk assessment |
|  | Desai,2009 | 46 | Not flood as a risk factor |
|  | Lau, 2010 | 7 | No data specific to flooding |
|  | Stern, 2010 | 47 | No data specific to flooding |
|  | Amilasan,2012 | 48 | Case description |
|  | Chen,2012 | 49 | No description on flood |
|  | Smith,2012 | 50 | Not sufficient data to extract |
|  | Bello,2013 | 51 | No description on flood |
|  | Miyazato, 2013 | 52 | No denominator |
|  | Cosson,2014 | 53 | Not sufficient data to extract |
|  | Munoz-Zanzi,2014 | 54 | Not flood as a risk factor |
|  | Kamath,2014 | 55 | No description on flood |
|  | Matono,2015 | 5 | Imported patient |
|  | Benacer, 2016 | 56 | No data specific to flooding |
|  | Dreyfus,2016 | 57 | Not including flooding related data |
|  | Lau, 2016 | 58 | No data specific to flooding |
|  | Fajriyah,2017 | 59 | Not sufficient data |
|  | Regmi,2017 | 60 | No description on flood |
|  | Matsushita,2018 | 61 | No data specific to flooding |
|  | Radi,2018 | 62 | No data specific to flooding |
|  | Wijerathne ,2018 | 13 | No denominator population |
