# Supplementary figures and images for: Risk factors for human leptospirosis following flooding: A meta-analysis of observational studies
Source: PLoS One. 2019 May 29;14(5):e0217643. doi: 10.1371/journal.pone.0217643 (PMC6541304; doi:10.1371/journal.pone.0217643)

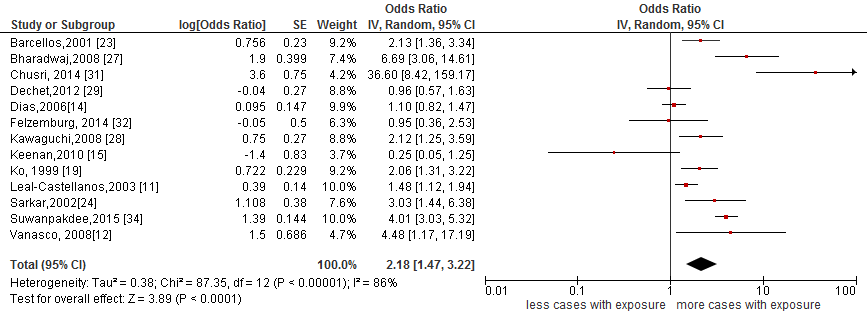

Supplement: S1 Fig — (TIF) [file pone.0217643.s004.tif]

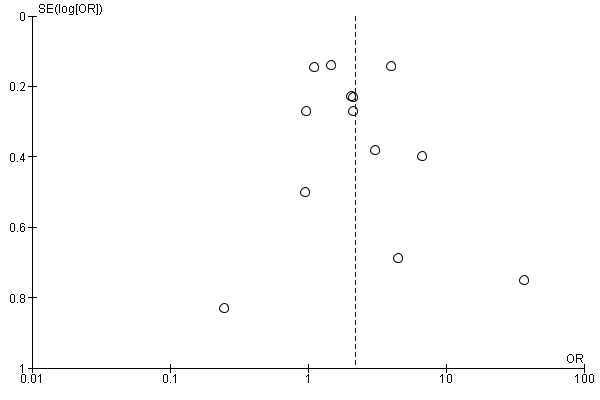

Supplement: S2 Fig — (TIF) [file pone.0217643.s005.tif]
